# Supplementary material for: Cathepsin B-Cleavable Cyclopeptidic Chemotherapeutic Prodrugs
Source: Molecules. 2020 Sep 18;25(18):4285. doi: 10.3390/molecules25184285 (PMC7570921; doi:10.3390/molecules25184285)
Supplement: Supplementary file 1 [file molecules-25-04285-s001.pdf]

Article

Cathepsin B-cleavable Cyclopeptidic Chemotherapeutic Prodrugs

Viktorija Herceg, Jordan Bouilloux, Karolina Janikowska, Eric Allémann and Norbert Lange

School of Pharmaceutical Sciences, Laboratory of Pharmaceutical Technology, ISPSO, University of Geneva, Rue Michel-Servet 1, Genève 4, CH-1211, Switzerland.

* Correspondence: norbert.lange@unige.ch

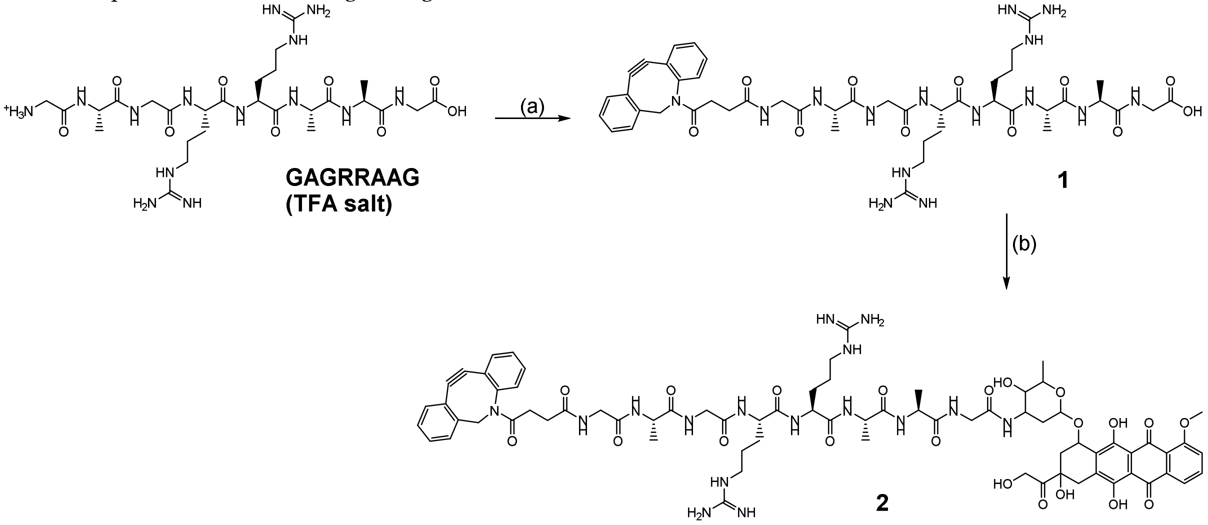

Scheme S1 - Generic synthesis pathway for the DBCO-GAGRRAAG-DOX conjugate. *Reagents and conditions*: (a) DBCO-NHS, DIPEA, argon (ar.), dark, room temperature (r. t.), overnight (o/n); (b) DOX (HCl salt), HATU, DIPEA, ar., dark, r. t., o/n; (c) DBCO-NH_2, HATU, DIPEA, ar., dark, r. t., o/n.

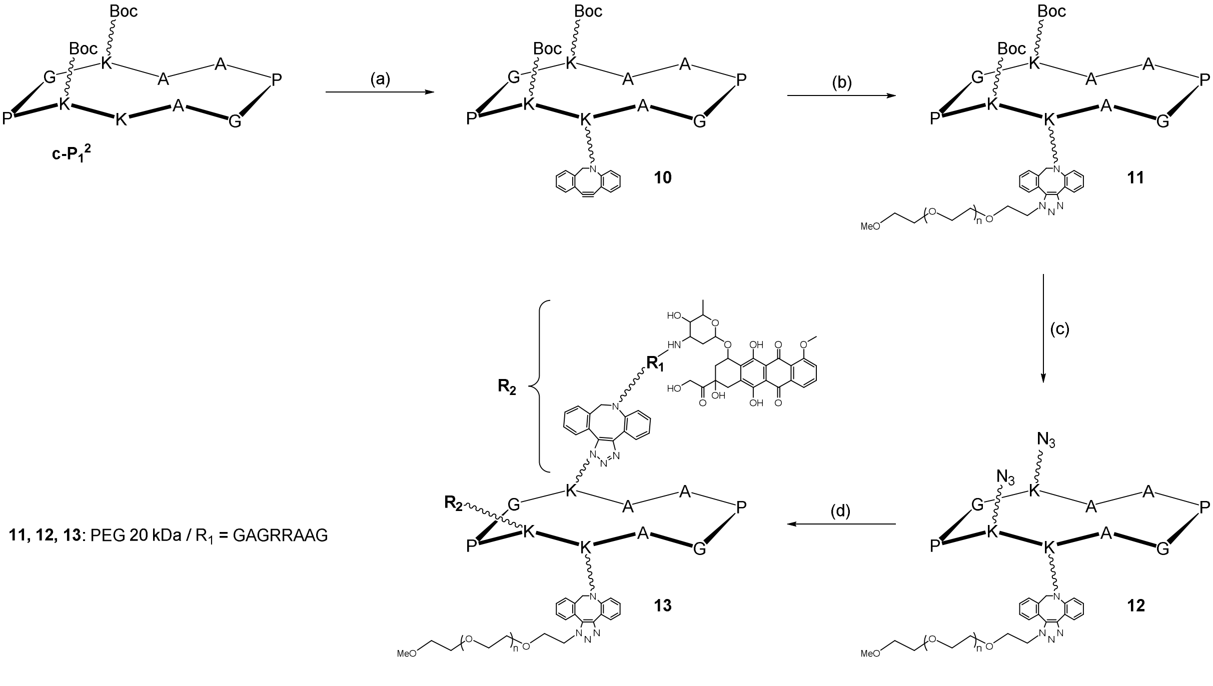

Scheme S2 - Generic synthesis pathway for the disubstituted-monoPEGylated conjugate $cPCP_{2/20}$. *Reagents and conditions*: (a) DBCO-NHS, argon (ar.), room temperature (r. t.), overnight (o/n); (b) α-azido-ω-methoxy-PEG (20 kDa), ar., r. t., o/n; (c) TFA/DCM (50:50), 30 min, r. t.; APA, DIPEA, HATU, ar., r. t., o/n; (d) **(2)**, ar., dark, r. t., o/n.

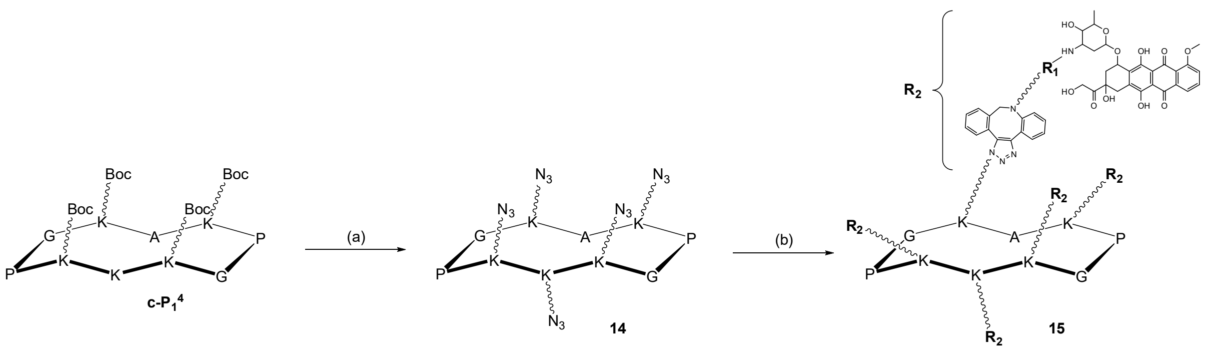

15: R_1 = GAGRRAAG

Scheme S3 - Generic synthesis pathway for the pentasubstituted conjugate cPCP5. *Reagents and conditions*: (a) TFA/DCM (50:50), 30 min, r. t.; azidoacetic acid-NHS, DIPEA, HATU, ar., r. t., o/n; (b) **(2)**, ar., dark, r. t., o/n.

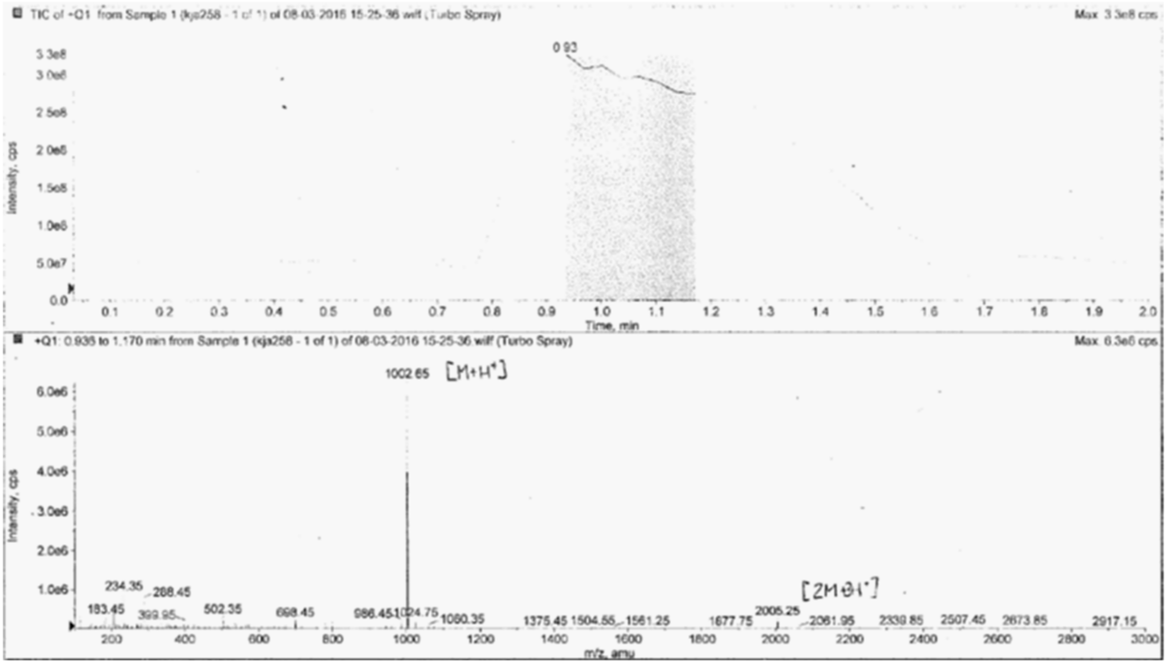

Figure S1 - ESI-MS analysis of DBCO-GAGRRAAG

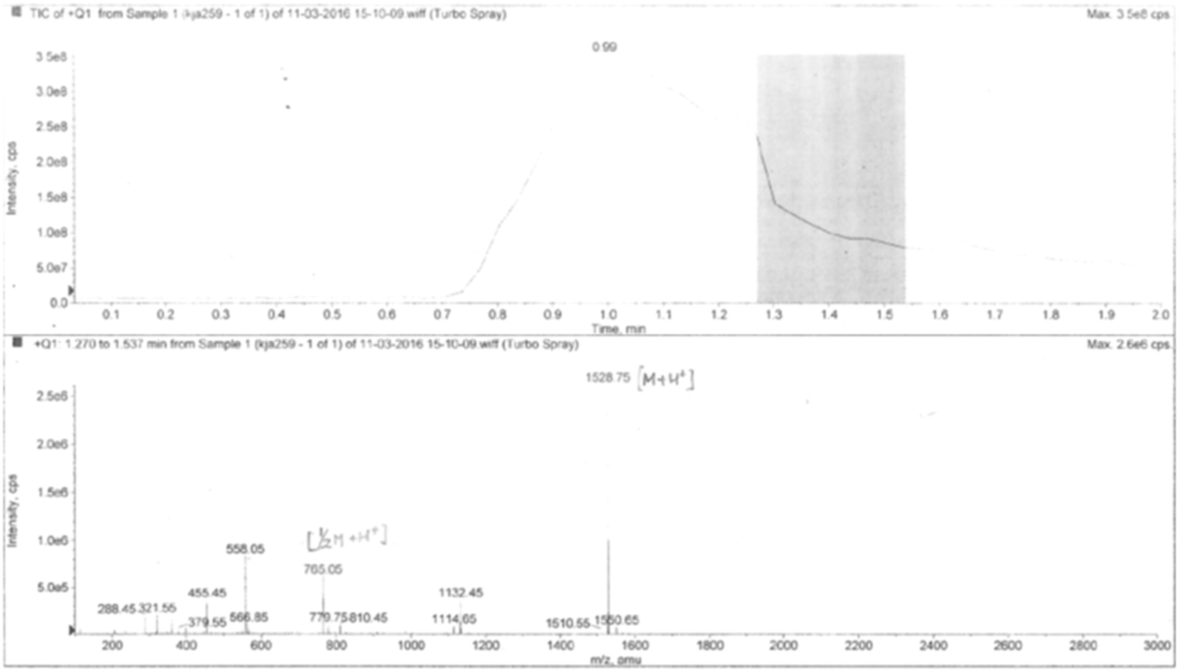

Figure S2 - ESI-MS analysis of DBCO-GAGRRAAG-DOX

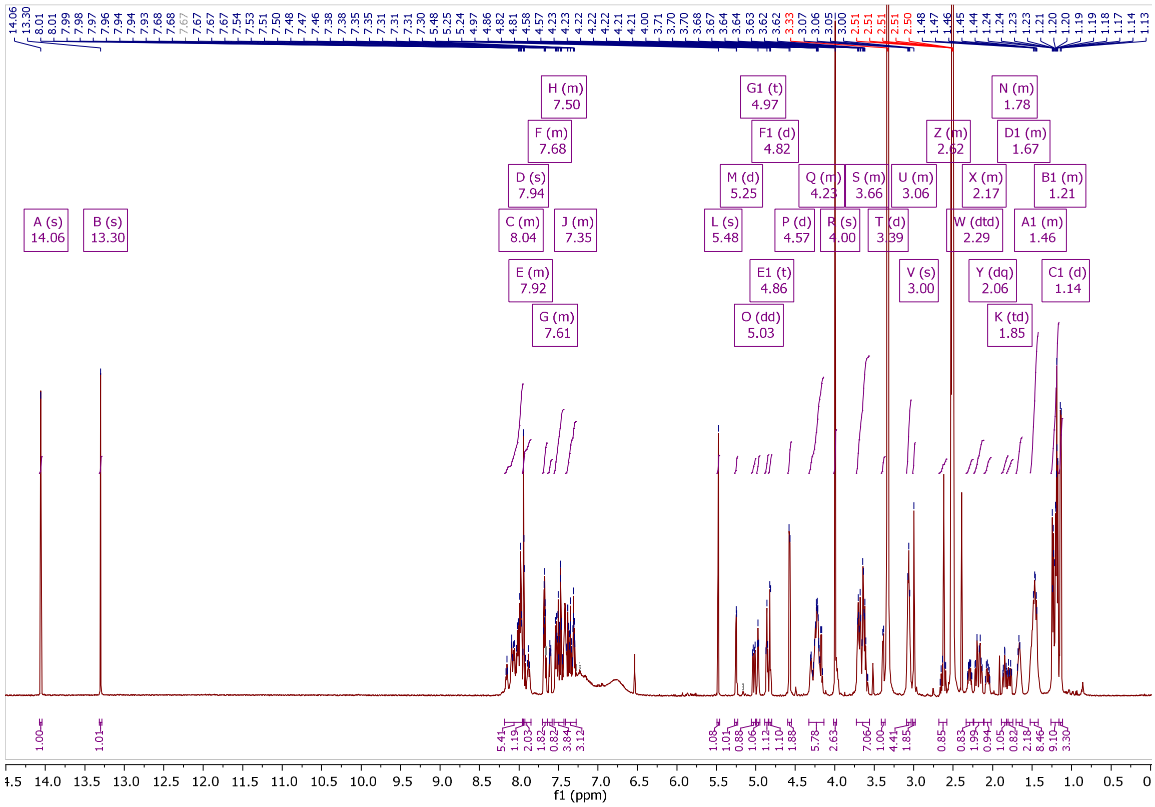

Figure S3 - ^{1}H NMR (DMSO-d6, 300 MHz) NMR analysis of DBCO-GAGRRAAG-DOX

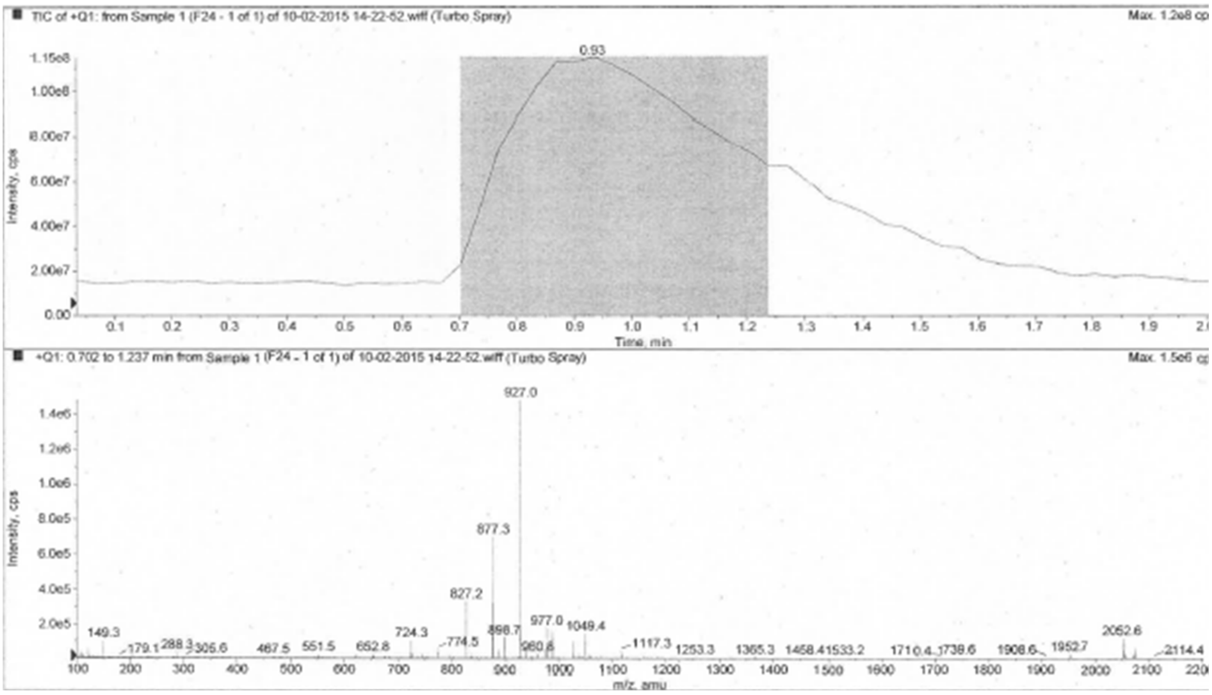

Figure S4 - ESI-MS analysis of $(DBCO)_2$-cP_2[4]

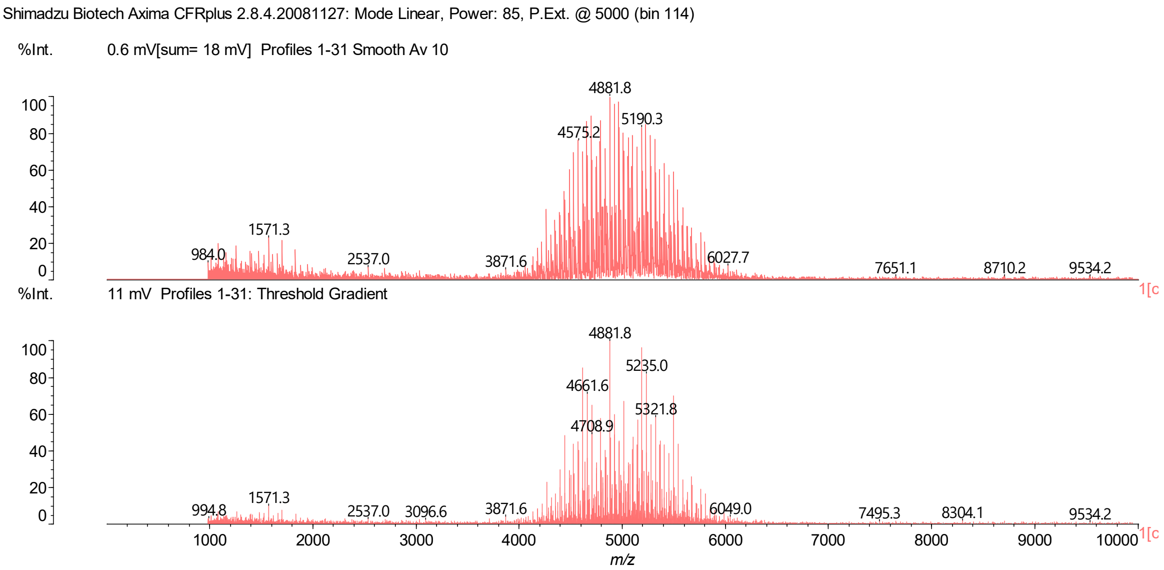

Figure S5 - MALDI-TOF analysis of MeO-PEG-N_3 5 kDa. Mw expected according to the supplier: 5079 Da

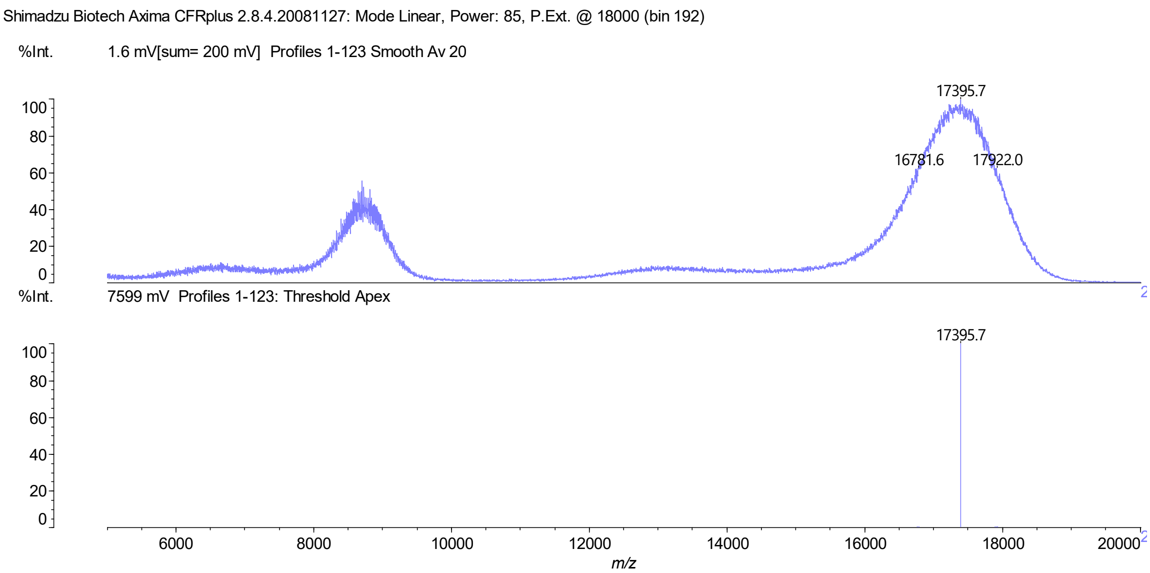

Figure S6 - MALDI-TOF analysis of $cPCP_{4/5}^{2}$

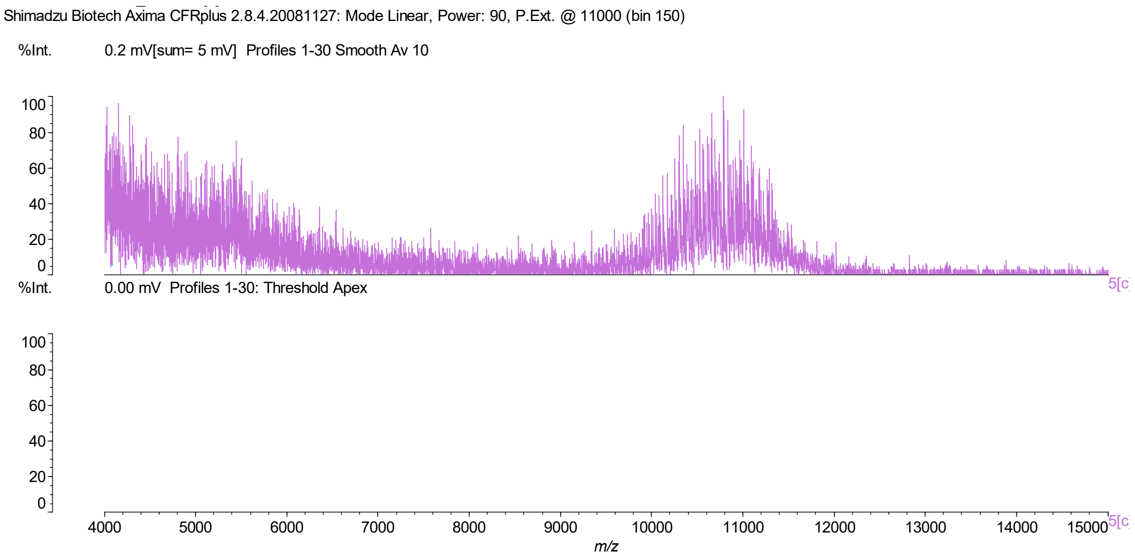

Figure S7 - MALDI-TOF analysis of MeO-PEG-N_3 10 kDa. Mw expected according to the supplier: 11153 Da; found: ~10800 Da.

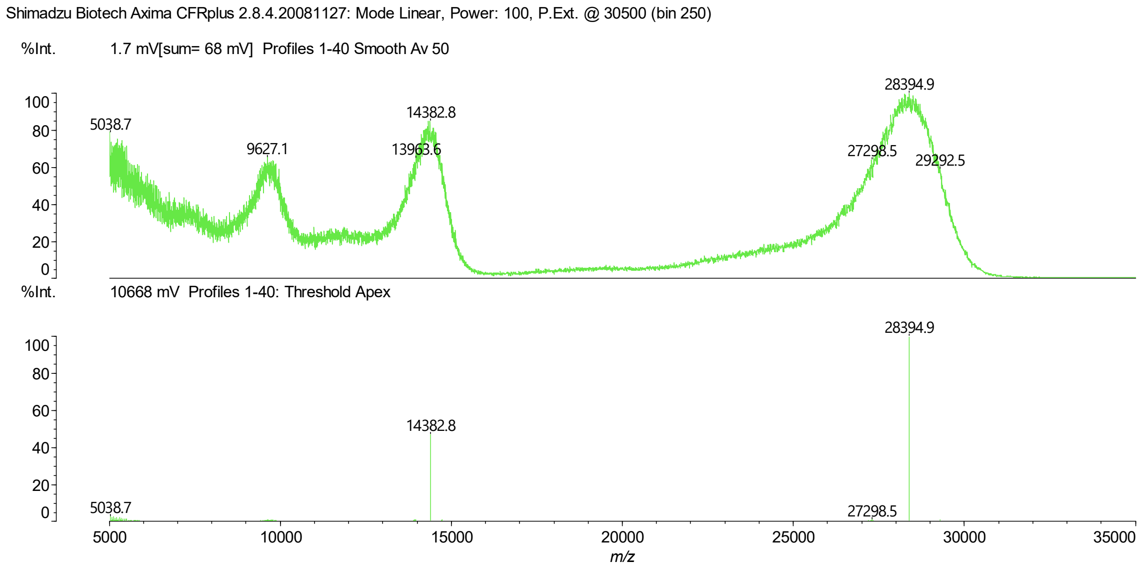

Figure S8 - MALDI-TOF analysis of $cPCP_{4/10}^{2}$.

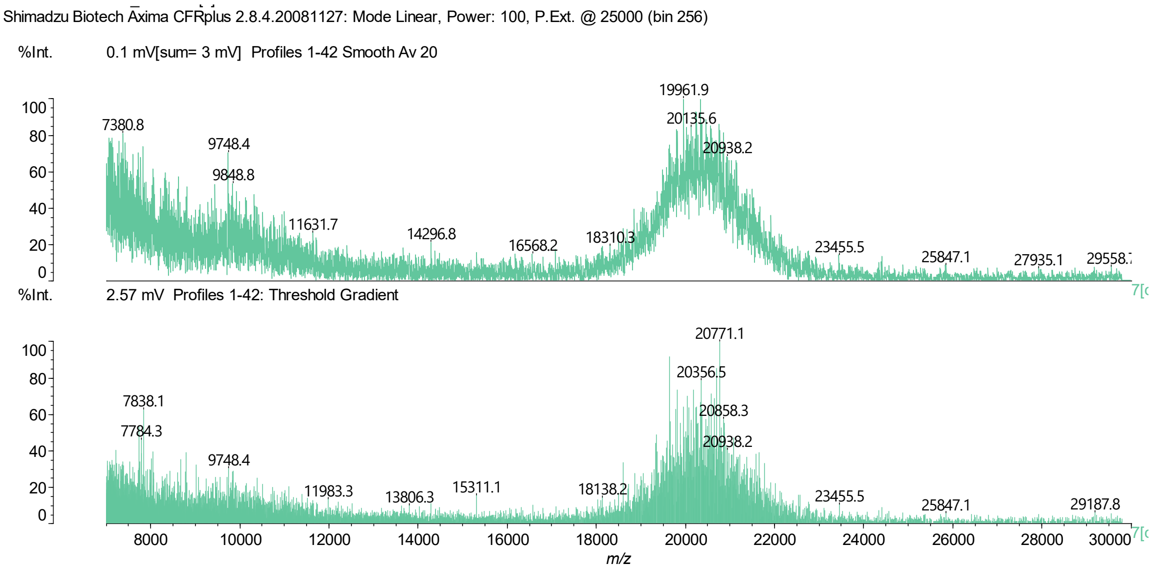

Figure S9 - MALDI-TOF of MeO-PEG-N_3 20 kDa. Mw expected according to the supplier: 22271 Da; found: ~20400 Da

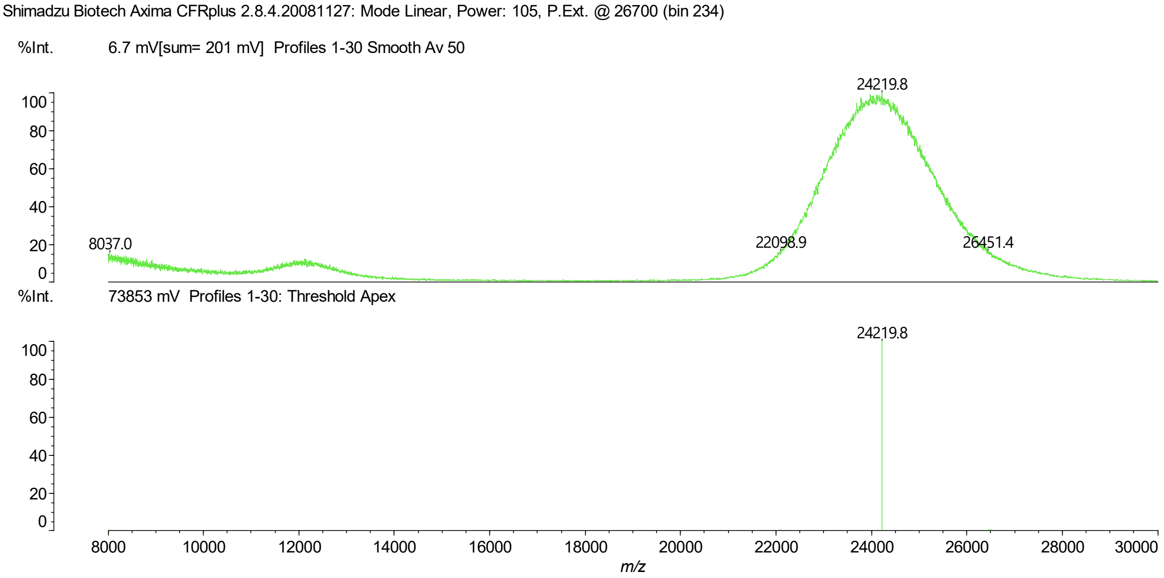

Figure S10 - MALDI-TOF analysis of $cPCP_{2/20}$

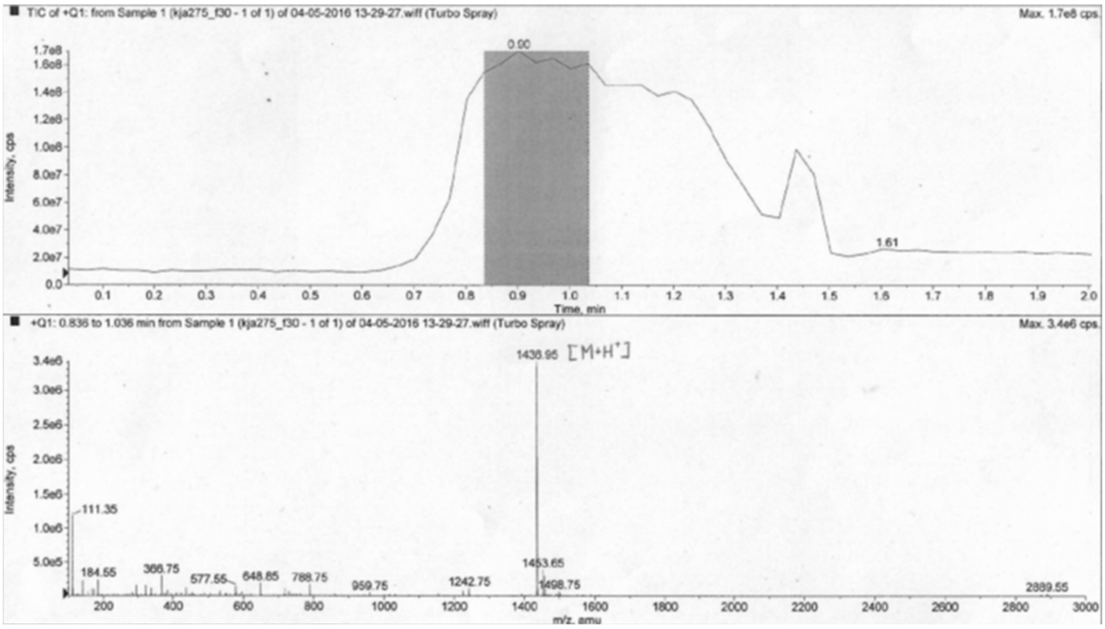

Figure S11 - ESI-MS analysis of cP_1^4 after Boc deprotection

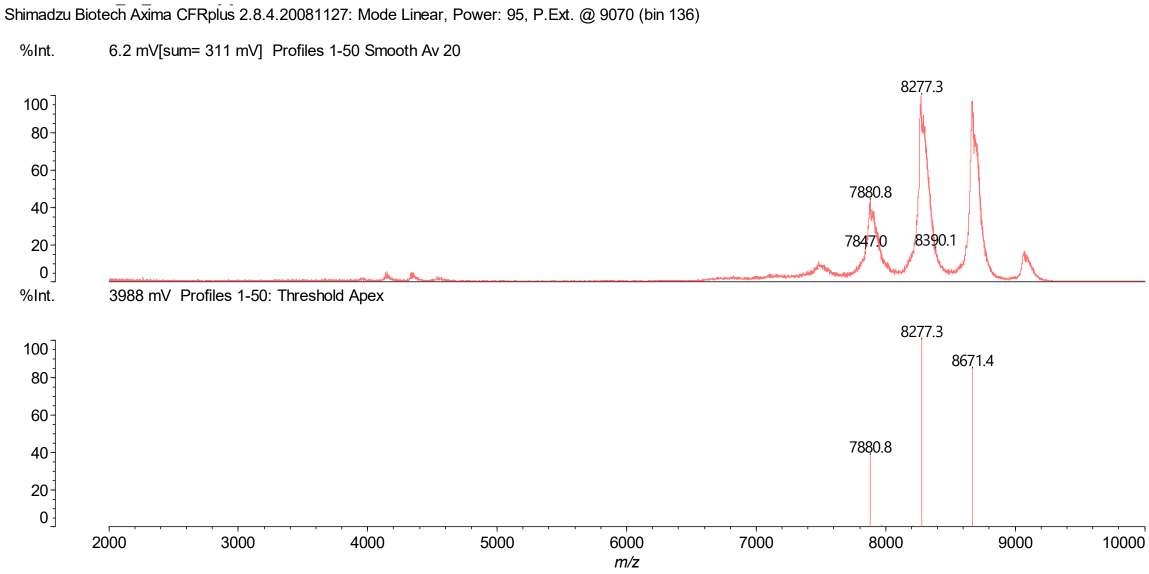

Figure S12 - MALDI-TOF analysis of $cPCP_5$.

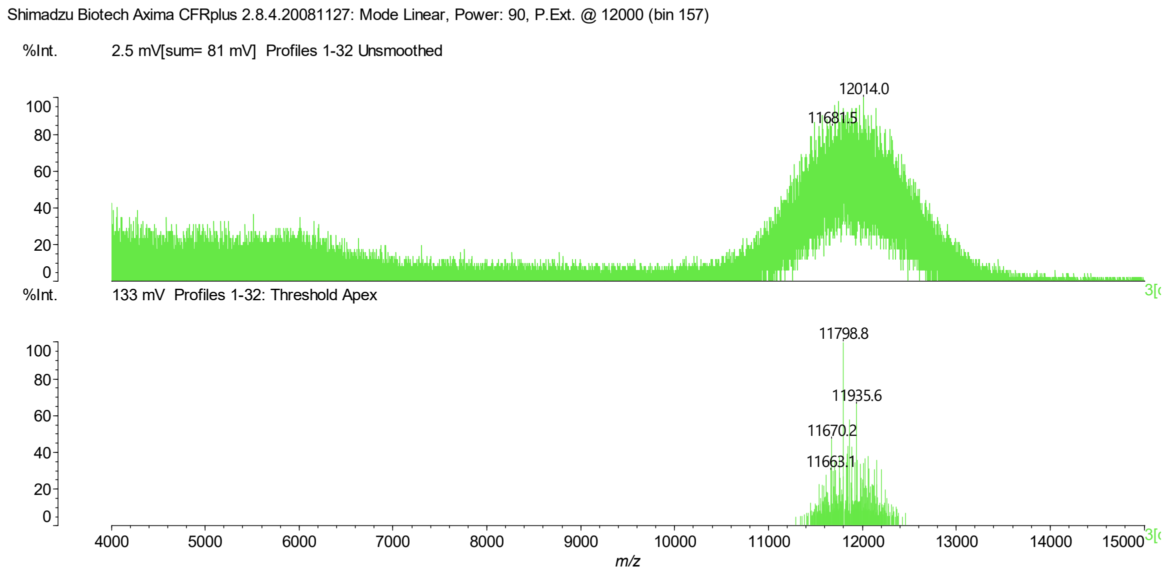

Figure S13 - MALDI-TOF analysis of PEG_{10}-GAGRRAAG-DOX

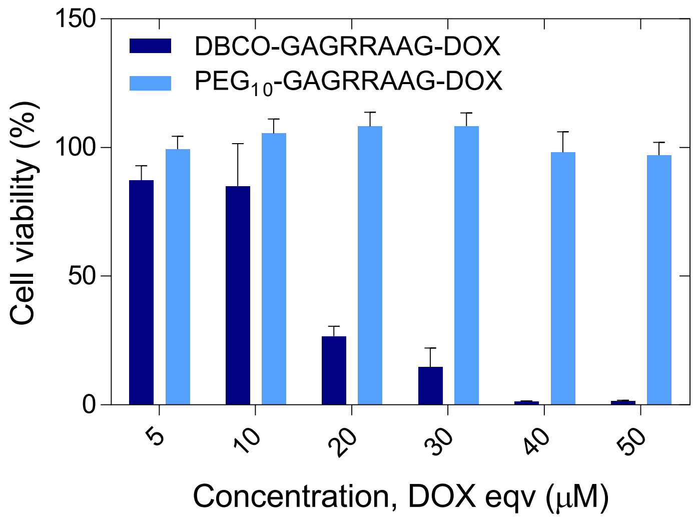

Figure S14 - WST-1 cell proliferation assay in HT1080 after 72 hours of incubation with PEG_{10}-GAGRRAAG-DOX (n = 3).

[Dox] mM	0.01	0.05	0.10	0.15	0.2	0.4	0.5	0.6	0.8	1.0
SR (%)	86.4±9.7	81.7±10.6	69.9±9.7	53.7±7.3	34.6±4.7	6.9±1.3	1.3±0.4	0.4±0.5	0±0.7	0.4±0.5

Table S1: WST-1 cell proliferation assay in HT1080 after 72 hours of incubation with DOX SR: Survival Rate

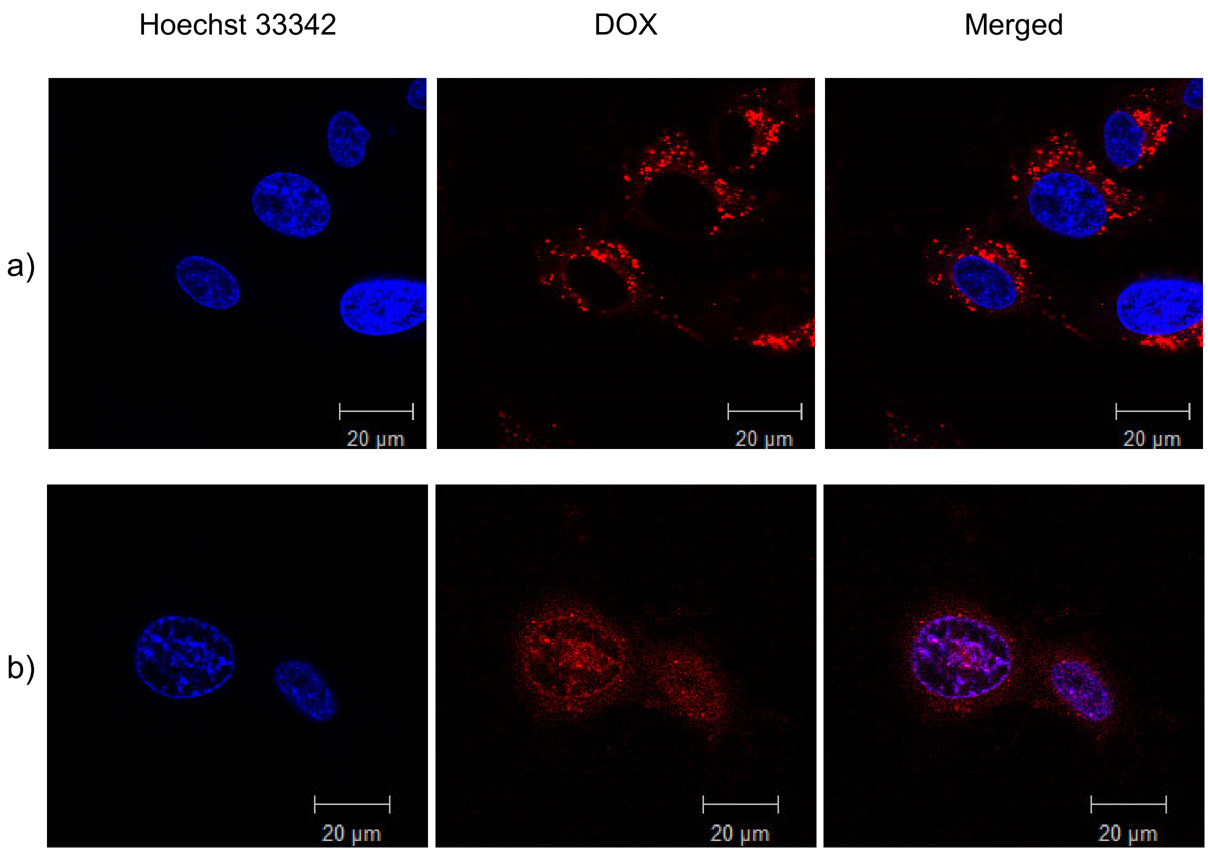

Figure S15 - HT1080 – fluorescence micrographs upon 24 hours of incubation with a) 30 µM of $cPCP_{4/5}^{2}$, b) 0.3 µM of free DOX. Magnification 63

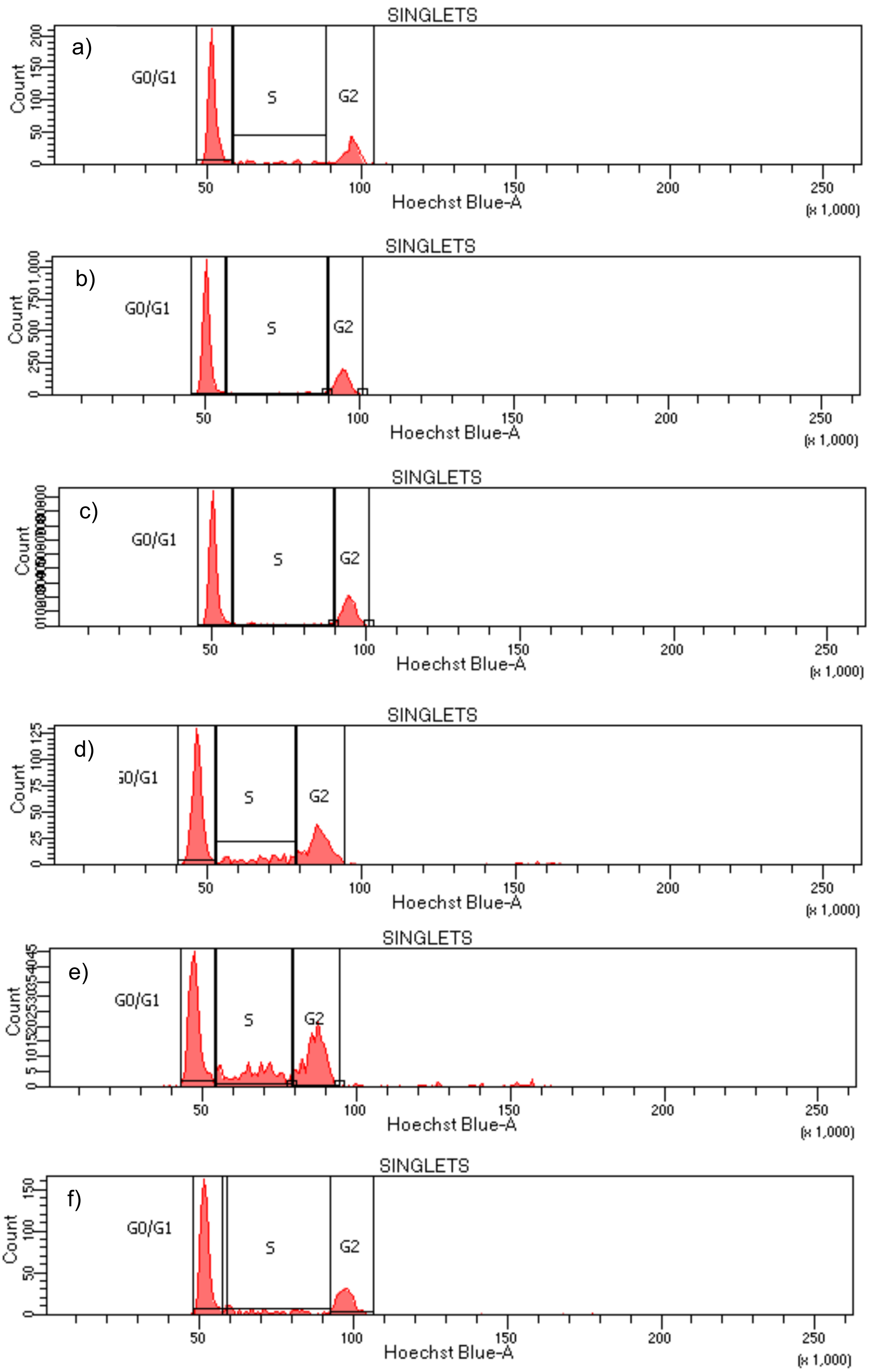

Figure S16 - HT1080-flow cytometry of cell cycle analysis after 24 hours of incubation with 0.3 μM (DOX eq) of a) $cPCP_{4/5}{}^{2}$, b) $cPCP_{4/10}{}^{2}$, c) $cPCP_{2/20}$, d) $cPCP_{5}$, e) free DOX and f) control.

Table S2 - Cell cycle analysis of HT1080 cells after 48 hours of incubation with equimolar concentration (DOX eq) of free DOX, $cPCP_{4/5}^2$ and $cPCP_5$.

Product name	*% G1*	*% S*	*% G2*
CTR	47.0 ± 1.7	25.3 ± 2.8	25.0 ± 1.7
DOX	29.2 ± 1.5	32.3 ± 4.4	33.6 ± 5.7
$cPCP_{4/5}^2$	50.2 ± 2.1	19.6 ± 10.4	30.2 ± 11.3
$cPCP_5$	35.45 ± 2.0	22.0 ± 4.9	39.9 ± 6.3

Table S3 - Cell cycle analysis of HT1080 cells after 72 hours of incubation with equimolar concentration (DOX eq) of free DOX, $cPCP_{4/5}^2$ and $cPCP_5$.

Product name	*% G1*	*% S*	*% G2*
CTR	45.6 ± 0.5	28.5 ± 2.1	20.9 ± 2.0
DOX	32.6 ± 3.2	35.8 ± 6.7	27.3 ± 8.1
$cPCP_{4/5}^2$	46.8 ± 1.3	28.9 ± 3.4	18.6 ± 2.6
$cPCP_5$	38.1 ± 3.7	19.4 ± 11.2	39.3 ± 8.7

a)

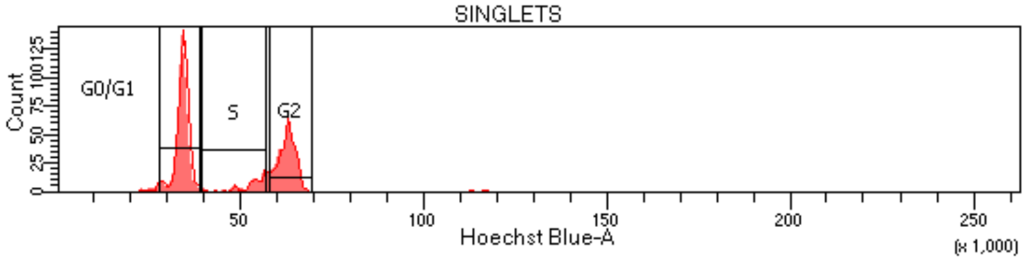

b)

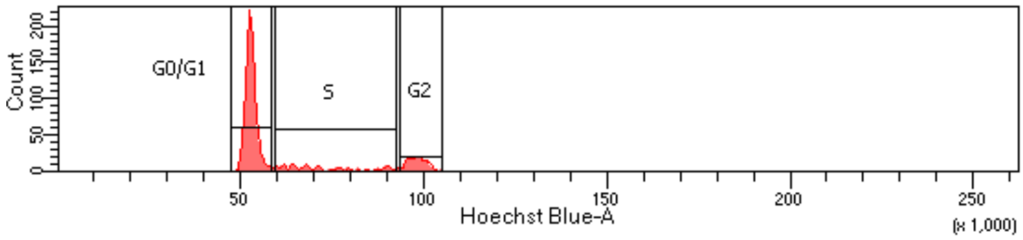

Figure S17 - HT1080 - flow cytometry of cell cycle analysis after 24 hours of incubation with A) 30 µM (DOX eq) of $cPCP_{4/5}^2$, B) control cells.
